# Effectiveness of Failure Mode and Effects Analysis in managing skin complications in ICU psychiatric patients: a real-world study

**DOI:** 10.3389/fpsyt.2025.1571858

**Published:** 2025-04-04

**Authors:** Ping Zhang, Haiyan Zhu, Yuanyuan Qian, Minmin Chen, Lin Liu

**Affiliations:** ^1^ Department of Intensive Care Unit, Nantong Fourth People’s Hospital, Nangtong, Jiangsu, China; ^2^ Department of Nursing, Jiangxi Provincial People’s Hospital, The First Affiliated Hospital of Nanchang Medical College, Nanchang, China

**Keywords:** psychiatric patients, intensive care unit (ICU), skin complications, Failure Mode and Effects Analysis (FMEA) method, wound care, risk management

## Abstract

**Objective:**

Psychiatric patients are particularly vulnerable to skin injuries, which can result in severe systemic complications and higher mortality rates. Therefore, improving skin wound management for ICU psychiatric patients through Failure Mode and Effects Analysis (FMEA) is crucial. This study aims to evaluate the effectiveness of FMEA in enhancing skin wound management in ICU settings, with a focus on identifying key risk factors and implementing targeted interventions to mitigate skin complications.

**Methods:**

A real-world study was conducted in the ICU of the Fourth People’s Hospital of Nantong, China, involving 615 psychiatric patients admitted between October 2022 and October 2024. Patients were divided into two groups: the control group received traditional wound care, while the observation group was managed using FMEA-based strategies. Key risk factors were evaluated through FMEA to prioritize interventions, Logistic regression analysis was used to assess the relationship between various risk factors and skin complications, helping to identify significant predictors of adverse skin events.

**Results:**

FMEA implementation led to a significant reduction in skin complications from 7.56% to 3.59% (*χ^2^ =* 4.69, *p* = 0.03). FMEA analysis identified key risk factors, including positioning management, skin hygiene, and nutritional support, with corresponding Risk Priority Numbers (RPN) calculated for each factor. Logistic regression analysis revealed that FMEA implementation was significantly associated with a reduced risk of skin complications (coefficients = -1.44, p = 0.01). Additionally, factors such as ADL, hypoalbuminemia, diabetes, and ICU length of stay were found to significantly influence the risk of skin complications (p < 0.01).

**Conclusion:**

FMEA is an effective tool for enhancing skin management practices and reducing skin complications in psychiatric ICU patients. Early identification of risk factors and the implementation of personalized skin care protocols can significantly improve patient outcomes and safety.

## 1 Introduction

### 1.1 Background

Mental disorders encompass a broad range of syndromes marked by significant disruptions in cognition, emotional regulation, or behavior, often resulting from dysfunction in psychological, biological, or developmental processes that underlie mental and behavioral functioning (1). Psychiatric patients face an increased risk of skin injuries, largely due to shear and friction forces, self-injurious behaviors, and the side effects of psychotropic medications (2, 3). These individuals are more vulnerable to developing skin wounds, which can arise from a variety of causes, including stupor, defiance, agitation, inability to perform activities of daily living, incontinence, refusal to eat, self-harm, and restricted mobility (4). Studies have reported a prevalence of 10% to 60% for the co-occurrence of psychiatric disorders and skin diseases (5–7).

Skin-related conditions, such as pressure ulcers and infections, significantly affect patient outcomes. These complications can lead to severe systemic infections, including sepsis, which contribute to pain, suffering, decreased quality of life, and increased morbidity and mortality (8, 9). Data suggest that the mortality rate for patients admitted with dermatological conditions is approximately 28.1%, higher than the general ICU mortality rate of around 20% (10).

### 1.2 Problem description

Effective skin management is crucial for psychiatric patients due to their increased risk of skin complications. Although conventional skin care practices have shown some efficacy, many patients continue to suffer from severe skin conditions, leading to adverse outcomes (11). Notably, most psychiatric patients are unable to self-report skin trauma. Therefore, it is essential that nursing professionals have a thorough understanding of skin wound prevention and possess the skills to accurately identify, assess, and manage various skin wounds (12). With the growing emphasis on improving the overall health and well-being of individuals with mental illnesses (13), there is an urgent need for further exploration and research into the management of skin injuries in psychiatric patients.

### 1.3 Available knowledge

While extensive research exists on wound care and management, studies specifically addressing skin care in Intensive Care Unit (ICU) psychiatric patients remain limited (12), especially regarding the application of Failure Mode and Effects Analysis (FMEA) in managing skin injuries within this population. FMEA is a systematic risk management tool designed to identify potential failures in processes and evaluate their effects (14). The methodology involves assessing the severity, occurrence, and detectability of failure modes to prioritize risks and implement corrective actions (15). Literature suggests that FMEA has been beneficial in the care of surgical patients (16, 17), stroke patients (18), cancer patients (19), burn patients (20), pediatric patients (21), and those with infections (22, 23).

### 1.4 Purpose

The literature indicates that FMEA is an effective intervention that enhances the professional capabilities of nurses. It offers an innovative approach to skin management in ICU psychiatric patients, enabling the implementation of targeted improvement strategies through quantitative assessments of potential risk factors before skin damage occurs.

## 2 Methods

### 2.1 Design

This study employs a prospective cohort design combined with FMEA to explore its application in skin wound management for ICU psychiatric patients. The aim is to systematically analyze the skin wound management process for psychiatric inpatients, identify potential risk factors, and implement targeted interventions using FMEA to enhance nursing skills and reduce the risk of skin wounds.

### 2.2 Setting

The study was conducted in the ICU of the Fourth People’s Hospital of Nantong, a 700-bed tertiary psychiatric hospital located in Jiangsu Province, China. The hospital serves as a leading medical center in the region. The study involved staff responsible for daily skin care and wound management, all of whom received training on the FMEA methodology and its application in skin management. The ward was equipped with standard resources for wound care.

### 2.3 Patient information and medical records

The electronic medical record system was utilized to review patients’ basic information and medical history, while nursing records from the ICU department provided details about skin injuries.

### 2.4 Participants

A total of 615 psychiatric patients admitted to the ICU of the hospital between October 2022 and October 2024 were enrolled in the study. Of these, 225 patients admitted from October 2022 to September 2023 formed the control group, while 390 patients admitted from October 2023 to September 2024 were included in the observation group.

Inclusion criteria: (1) Patients confirmed to be critically ill and requiring ICU care. (2) Patients aged ≥18 years. (3) Patients who voluntarily signed the informed consent.

Exclusion criteria: (1) Patients with pre-existing skin conditions at the time of enrollment. (2) Patients with an expected survival time of less than 3 months. (3) Patients or family members who voluntarily withdrew from the study or treatment.

### 2.5 Interventions

Two distinct wound care approaches were implemented: the traditional wound care method (control group) and the FMEA-based wound care method (observation group). The interventions aimed to evaluate the effectiveness of the FMEA approach in improving skin wound management in psychiatric ICU patients.

#### 2.5.1 Traditional wound care method (control group)

In the control group (225 patients, October 2022 - September 2023), traditional wound care methods were employed. These methods included an initial risk assessment for common skin conditions, considering factors such as the presence and severity of incontinence, skin condition, and nutritional status. Based on the assessment, preventive measures were implemented for patients with varying risk levels, following the principles of “cleanse, moisturize, protect.” If skin complications occurred, symptomatic treatments, such as antibiotics, antifungal medications, and ostomy powder, were provided, while ensuring that the skin remained dry throughout the treatment process (24).

#### 2.5.2 FMEA-based wound care method (observation group)

In the observation group (390 patients, October 2023 - September 2024), the FMEA methodology was introduced to enhance wound care practices. The FMEA approach aimed to identify and address the underlying causes of skin complications in psychiatric ICU patients through systematic risk analysis and the implementation of targeted interventions.

A FMEA team, consisting of doctors, nurses, and specialized medical staff from the hospital’s infection prevention department, was formed after undergoing comprehensive training on the FMEA methodology. The team regularly held seminars to discuss and review factors influencing skin complications in psychiatric patients (25). Eight primary categories of risk factors were identified: Initial Assessment, Positioning Management, Skin Hygiene, Nutritional Support, Catheter Management, Monitoring for Infection, Skin Dressing, and Functional Exercise, as shown in **Table 1**.

**Table 1:** Key failure modes, causes, effects, and RPN prioritization in psychiatric ICU patients.

Step	Failure Mode	Causes	Effects	Current Controls	RPN (S/O/D)	Action Plan
**Initial Assessment**	Incomplete or delayed skin assessment	High workload, lack of training, patient agitation, low mobility, comorbid diseases such as diabetes, etc	Missed early signs of skin breakdown, hyperglycemia	Staff training, behavioral tools, blood sugar monito	8/6/4 = 192	Implement routine skin assessments within 12-hour intervals, With the support of an endocrinologist, a glucose-lowering treatment plan was developed.
**Positioning Management**	Pressure from prolonged positioning	Sedation, restraints, lack of awareness of proper positioning	Pressure ulcers, skin breakdown, discomfort	Turning schedules, special mattresses, repositioning protocols	9/7/3 = 189	Create a standardized repositioning protocol; enforce schedule adherence.
**Skin Hygiene**	Poor skin hygiene & moisture management	Lack of awareness, inadequate products, insufficient staff supervision	Skin irritation, infection, increased risk of breakdown	Skin care guidelines, moisture barriers, routine checks	9/6/3 = 162	Provide targeted training on skin care protocols and use of barrier creams.
**Nutritional Support**	Insufficient nutrition affecting skin health	Inadequate food intake, poor adherence to nutrition plan, low immunity	Impaired skin regeneration, delayed healing	Nutritional assessment, supplements, diet monitoring	7/6/4 = 168	Develop a nutrition improvement plan with daily monitoring and support from dieticians.
**Catheter Management**	Prolonged catheter retention	Delayed replacement, lack of education on catheter care	Skin irritation, urinary tract infections (UTIs), sepsis	Timely catheter checks, replacement protocols, education	9/5/4 = 180	Set up alarms for catheter checks; provide regular catheter care training for staff.
**Monitoring for Infection**	Failure to detect infection early	Missed non-verbal cues, inadequate staff awareness, low immunity	Sepsis, worsening infection, skin breakdown	Monitoring protocols, infection screening, non-verbal cue training	9/5/4 = 180	Implement real-time infection monitoring; enhance staff training on non-verbal signs of infection.
**Skin Dressing**	Dressing removed prematurely or incorrectly	Patient agitation, lack of proper dressing techniques, poor material choice	Infection, delayed healing, skin irritation	Tamper-proof dressings, proper application techniques	8/6/3 = 144	Develop and enforce standards for dressing application; offer training on dressing techniques.
**Functional Exercise**	Lack of participation in mobility exercises	Insufficient guidance, low patient motivation, mobility restrictions	Muscle atrophy, skin breakdown due to immobility	Physical therapy involvement, patient education	7/6/3 = 126	Increase patient engagement with targeted functional exercises.

RPN, Risk Priority Number; O, Likelihood; S, Severity; D, Detectability.

The FMEA team identified 8 major risk factors for ICU psychiatric patients, including initial assessment, posture management, skin hygiene, nutritional support, catheter management, infection monitoring, skin dressing and functional exercise. The failure modes, causes, effects and current control of risk factors were analyzed in team. Finally, the RPN was calculated according to O, S, D, and the RPN was calculated. The action plan was prioritized based on the RPN results.

Each identified risk factor was evaluated using three key components: Likelihood (O), Severity (S), and Detectability (D), rated on a scale from 1 to 10. The Risk Priority Number (RPN) was calculated using the formula: RPN = O × S × D. This resulted in a score range from 0 to 1000, helping to prioritize the most critical risks, as shown in **Table 1**.

Based on the FMEA analysis, tailored improvement plans were developed for high-risk events, addressing specific patient needs to improve patient outcomes, reduce complications, and optimize the care process, as shown in **Table 1**.

### 2.6 Research variables

#### 2.6.1 Dependent variables

Skin injuries commonly observed in psychiatric patients include pressure ulcers, diabetic ulcers, vascular ulcers, burns, and self-inflicted skin trauma (2, 26). In this study, the occurrence of pressure ulcers, skin ulcers, and skin injuries will be monitored before and after the implementation of FMEA. (1) Pressure Ulcers: Defined according to the National Pressure Ulcer Advisory Panel (NPUAP) 2016 guidelines (27). (2) Skin Ulcers: Includes vascular ulcers, neuropathic ulcers, infectious ulcers, and pressure ulcers (28). (3) Skin Trauma: Refers to damage to the skin and subcutaneous tissue caused by injuries other than cuts, lacerations, or stab wounds (29).

#### 2.6.2 Control variables

Based on FMEA results, previous studies, and data availability, the following covariates were identified: socio-demographic factors (gender, age, marital status), health status factors (BMI, hypoalbuminemia, diabetes, activities of daily living [ADL]), and treatment-related factors (diagnosis, ICU length of stay, and whether surgery was performed). These factors are known to influence the risk of developing skin lesions (30, 31). Relevant data was extracted from the hospital’s medical record system and nursing records. BMI was categorized according to the Chinese hygiene standard for adult weight classification: Normal (18.5 kg/m² ≤ BMI < 24.0 kg/m²), Underweight (BMI < 18.5 kg/m²), and Overweight or Obese (BMI ≥ 24.0 kg/m²) (32). ADL was assessed based on functional abilities required for bed mobility (positioning), ambulation, toileting, and eating, representing four levels: Never dependent, Sometimes dependent, Usually dependent, and Always dependent (33). Relevant diagnoses were based on the ICD-10 (International Classification of Diseases 10th Revision) Chapter V, which covers mental and behavioral disorders. Hypoalbuminemia was defined as a serum total albumin level <60 g/L or an albumin level <30 g/L (34), while diabetes was defined as fasting blood glucose ≥7.0 mmol/L (35).

### 2.7 Data analysis

Statistical analysis was performed using Stata 16 (StatCorp USA). Continuous variables were presented as means with standard deviations, and comparisons between groups were made using independent sample t-tests. Categorical data were expressed as proportions and percentages, with comparisons conducted using chi-square tests. A logistic regression model was used to assess the impact of FMEA and other factors on skin management for ICU psychiatric patients.

## 3 Results

### 3.1 Demographic and clinical characteristics

Of the 615 psychiatric patients enrolled between October 2022 and October 2024, 225 patients were in the control group, and 390 were in the observation group. The average age of participants was 44.33 ± 9.87 years. The demographic and clinical characteristics between the two groups were not significantly different (p > 0.05), as shown in **Table 2**.

**Table 2:** Characteristics of ICU psychiatric patients.

Characteristic	Total (n=615)	Control group (n=225)	Observation group (n=390)	*t/χ^2^*	*p*
Dermatologic, n (%)					
Yes	31 (5.04)	17 (7.56)	14 (3.59)	4.69	0.03
No	584 (94.96)	208 (92.44)	376 (96.41)		
Age, Mean ± SD	44.33 ± 9.87	43.42 ± 13.84	44.85 ± 6.53	-1.74	0.08
Sex, n (%)					
Male	400 (34.96)	86 (38.22)	261 (33.08)	1.66	0.20
Female	215 (65.04)	139 (61.78)	129 (66.92)		
Partner, n (%)					
Have partner	191 (31.06)	64 (28.44)	127 (32.56)	1.13	0.29
Sigle	424 (68.94)	161 (71.56)	263 (67.44)		
BMI, n (%)					
Normal	96 (15.61)	38 (16.89)	58 (14.87)	0.79	0.67
Underweight	256 (41.63)	89 (39.56)	167 (42.82)		
Overweight or Obese	263 (42.76)	98 (43.56)	165 (42.31)		
ICD, n (%)					
F00-F09	50 (8.13)	15 (6.67)	35 (8.97)	7.04	0.13
F10-F19	91 (14.80)	29 (12.89)	62 (15.90)		
F20-F21	68 (11.06)	18 (8.00)	50 (12.82)		
F30-F22	280 (45.53)	112 (49.78)	168 (43.08)		
F40-F43	126 (20.49)	51 (22.67)	75 (19.23)		
ADL, n (%)					
Never dependent	409 (66.50)	157 (69.78)	252 (64.62)	2.83	0.42
Sometimes dependent	132 (21.46)	43 (19.11)	89 (22.82)		
Usually dependent	38 (6.18)	15 (6.67)	23 (5.90)		
Always dependent	36 (5.85)	10 (4.44)	26 (6.67)		
Surgery, n (%)					
Yes	428 (69.59)	157 (69.78)	271 (69.49)	0.01	0.94
No	187 (30.41)	68 (30.22)	119 (30.51)		
Hypoproteinemia, n (%)					
Yes	44 (7.15)	14 (6.22)	30 (7.69)	0.46	0.50
No	571 (92.85)	211 (93.78)	360 (92.31)		
Diabetes, n (%)					
Yes	55 (8.94)	17 (7.56)	38 (9.74)	0.84	0.36
No	560 (91.06)	208 (92.44)	352 (90.26)		
ICU Stay, Mean ± SD	14.94 ± 10.26	15.29 ± 10.92	14.74 ± 9.87	0.65	0.52

BMI, Body Mass Index; ICD, International Classification of Diseases; ADL, Activities of Daily Living; ICU, Intensive Care Unit.

### 3.2 Impact of FMEA on skin complications

The prevalence of skin disease complications among ICU psychiatric patients in the study was 5.04%. After the implementation of FMEA, there was a significant reduction in skin complications, decreasing from 7.56% prior to FMEA to 3.59% afterward (*χ^2^ =* 4.69, p = 0.03), as shown in **Table 2**.

### 3.3 Factors influencing skin complications

Multivariate logistic regression analysis revealed that FMEA implementation was significantly associated with a reduced risk of skin complications (coefficients = -1.44, p = 0.01). In addition, factors such as the ADL, hypoalbuminemia, diabetes, and ICU length of stay were significantly associated with skin complications. Specifically, compared to patients who were fully independent, those who were usually dependent or always dependent had a significantly higher risk of skin complications (coefficients = 3.47 and 2.88, p < 0.01). Patients with hypoalbuminemia had a significantly higher risk of skin complications compared to those with normal albumin levels (coefficient = 2.00, p < 0.01), and similarly, patients with diabetes had a significantly higher risk than those with normal blood glucose levels (coefficient = 2.00, p < 0.01). Furthermore, the longer the ICU stay, the higher the risk of skin complications (coefficient = 0.06, p = 0.01), as shown in **Table 3**.

**Table 3:** Factors associated with dermatologic conditions in ICU psychiatric patients.

	Coefficient	Standard Error	95% Confidence Interval		z	P>z
Group						
Control	Ref.					
Observation	-1.44	0.58	-2.57	-0.31	-2.49	0.01
Age	0.03	0.03	-0.02	0.09	1.24	0.21
Sex						
Female	Ref.					
Male	-0.62	0.63	-1.85	0.61	-1.00	0.32
Partner						
Sigle	Ref.					
Have partner	0.31	0.82	-1.30	1.92	0.38	0.71
BMI						
Normal	Ref.					
Underweight	0.16	1.30	-2.38	2.71	0.13	0.90
Overweight or Obese	1.47	1.31	-1.10	4.04	1.12	0.26
ICD						
F00-F09	Ref.					
F10-F19	-0.98	1.28	-3.49	1.53	-0.77	0.44
F20-F21	-0.69	1.50	-3.63	2.25	-0.46	0.65
F30-F22	-0.47	1.20	-2.82	1.87	-0.40	0.69
F40-F43	0.74	1.25	-1.71	3.18	0.59	0.56
ADL, n(%)						
Never dependent	Ref.					
Sometimes dependent	-0.14	1.20	-2.50	2.22	-0.12	0.91
Usually dependent	3.47	0.91	1.69	5.24	3.82	0.00
Always dependent	2.88	0.98	0.96	4.79	2.94	0.00
Surgery						
No	Ref.					
Yes	-0.11	0.67	-1.43	1.21	-0.16	0.87
Hypoproteinemia						
No	Ref.					
Yes	2.00	0.67	0.69	3.31	2.99	0.00
Diabetes						
No	Ref.					
Yes	1.84	0.80	0.28	3.41	2.31	0.02
ICU Stay	0.06	0.02	0.01	0.11	2.53	0.01

BMI, Body Mass Index; ICD, International Classification of Diseases; ADL, Activities of Daily Living; ICU, Intensive Care Unit.

## 4 Discussion

The results of this study demonstrate that the implementation of FMEA significantly reduced the incidence of skin complications among ICU psychiatric patients, emphasizing its potential as an effective quality improvement tool for managing patient safety and clinical outcomes. Furthermore, this study identified several risk factors, such as ADL score, hypoalbuminemia, diabetes, and ICU length of stay, which provide a basis for preventing skin complications in ICU psychiatric patients.

FMEA revealed potential failure modes in the skin care process, highlighting critical issues such as delayed infection detection, inadequate preventive measures, and improper body management. These issues are particularly significant in psychiatric ICU patients, who often suffer from multiple comorbidities such as prolonged immobility, malnutrition, and cognitive impairments, making them more prone to skin lesions (36). FMEA helped us identify these high-priority risks and provided a systematic approach to improve nursing processes, thereby reducing the incidence of skin complications (23, 37).

The regression analysis confirmed that ADL score, hypoalbuminemia, diabetes, and ICU length of stay were significant risk factors for dermatologic conditions in ICU psychiatric patients. These factors were directly or indirectly related to the occurrence of skin complications in ICU psychiatric patients identified by FMEA. By analyzing the causes and developing corresponding action plans, the occurrence of skin complications can be significantly reduced. Specifically, ADL-dependent patients, due to their limited ability to perform activities independently, experience prolonged pressure on the skin (38), which is directly related to “Positioning Management and Functional Exercise” identified by FMEA. Through FMEA-driven dynamic care plans, the risk of skin damage is significantly reduced (39).

The findings also highlight the importance of managing comorbid conditions such as diabetes and hypoalbuminemia. Microcirculation disorders caused by diabetes mellitus and malnutrition caused by hypoalbuminemia can lead to delayed skin wound healing in ICU psychiatric patients (23, 40). The findings of this study are consistent with prior reports on the interaction between skin conditions and psychological comorbidities, suggesting the need for integrated management of metabolic and skin health (40). FMEA further guided the implementation of enhanced blood glucose monitoring for diabetic patients and nutrition improvement plans, with daily monitoring and support from dieticians (36).

The length of stay in the ICU was positively correlated with skin complications, and indirectly correlated with failure modes such as “Skin Hygiene,” “Catheter Management,” “Monitoring for Infection,” and “Skin Dressing,” as identified by FMEA (38). Similarly, a meta-analysis supported the potential benefits of process optimization in shortening ICU stay duration (36).

## 5 Strengths and limitations

### 5.1 Strengths

This study provides valuable insights into the impact of FMEA on the reduction of skin complications in ICU psychiatric patients, a population often underrepresented in quality improvement research in critical care settings. One of the key strengths of this study is its real-world design, providing robust evidence. The inclusion of a large sample size (615 patients) further strengthens the generalizability and robustness of the results. Additionally, the multivariate logistic regression analysis allowed for the control of potential confounders, such as ADL scores, comorbidities, and ICU length of stay, providing a clearer understanding of the factors contributing to skin complications in this population.

Another strength of the study is its focus on the psychiatric ICU population, which is often overlooked in skin complication research. By specifically targeting this group, the study addresses an important gap in the literature and contributes to a broader understanding of the unique risks and challenges faced by psychiatric patients in the ICU. The identification of key risk factors, such as hypoalbuminemia and diabetes, provides practical insights that can be used to tailor interventions and improve patient care in this high-risk group.

### 5.2 Limitations

Despite the strengths of this study, several limitations should be acknowledged. First, the observational design means that while we were able to assess the association between FMEA implementation and skin complications, causality cannot be definitively established. Although the significant reduction in skin complications following FMEA implementation is promising, other unmeasured factors, such as changes in ICU staffing or patient care protocols, may have also contributed to the observed improvement.

Another limitation is that, although the FMEA analysis identified key failure modes, the effectiveness of the early warning skin management strategies that were implemented has yet to be fully evaluated. Future studies should assess the long-term impact of these interventions on patient outcomes, including the prevention of skin lesions, as well as the associated healthcare costs of skin care in ICU settings.

Additionally, while our study included a large sample size, it was conducted at a single institution, which may limit the generalizability of the findings. Future multicenter studies are needed to validate these findings and explore additional risk factors, such as skin perfusion and moisture levels, which were not sufficiently assessed in this study.

## 6 Conclusion

In conclusion, this study identifies several significant risk factors for dermatologic conditions in critically ill psychiatric patients, including ADL dependency, hypoalbuminemia, diabetes, and ICU length of stay. These findings emphasize the importance of early identification and intervention for at-risk patients, as well as the implementation of personalized skin care protocols. Through a multidisciplinary approach that incorporates preventive measures, education, and monitoring, healthcare teams can significantly reduce the incidence of skin complications and improve patient outcomes in the ICU. FMEA plays a crucial role in reducing ICU psychiatric patients’ complications by identifying key risk factors and optimizing nursing care processes, ensuring better patient safety and skin health.

## Data Availability

The raw data supporting the conclusions of this paper are provided by the Fourth People’s Hospital of Nantong City, Jiangsu Province, China. Further inquiries need to apply to hospital for approval. Research data are not shared. Requests to access the datasets should be directed to LL, liulin2025@126.com.
